# Left Sided Gastroschisis

**DOI:** 10.21699/jns.v6i2.500

**Published:** 2017-04-15

**Authors:** Sandip Kumar Rahul, Ramdhani Yadav, Vijayendra Kumar, Vinit K Thakur, Zaheer Hasan, Apurva Agarwal

**Affiliations:** DepartmentDepartment of Pediatric Surgery, Indira Gandhi Institute of Medical Sciences, Patna, India

**Dear Sir**

Abdominal wall defect in Gastroschisis is usually located to the right of the umbilical cord. We discuss a rare case of left-sided gastroschisis. A 3-day-old female, borne of caesarean delivery at term to a 20year-old mother, came to the emergency with bowel loops lying outside the abdominal cavity. No significant antenatal history was present. Antenatal sonogram done outside the hospital at 28 weeks gestation had detected the lesion. On examination, this 2 KG child was lethargic and febrile. Matted and oedematous small bowel loops without any covering membrane were lying outside the abdominal cavity through a defect of approximate size 2.5x2.5cm, to the left of the umbilical cord (Fig.1). There was a perforation in the exposed bowel through which meconium came out. Routine blood investigations revealed leukocyte count of 13800. At surgery, the defect was extended for 2cm on either side vertically. The eviscerated bowel loops were meticulously separated to reach the site of perforation in the proximal ileum. There were multiple atretic segments in the distal bowel. Abdominal wall was manually stretched before returning the bowel loops inside the abdomen and fashioning a stoma at the site of perforation. Patient had stormy postoperative course and died on the 5th postoperative day due to sepsis-related complications.

Left sided gastroschisis (LSG) is rare with only 23 reported cases in literature. [1, 2] Most of these cases have been females. Compared to the commonly observed right sided lesions, LSG have increased incidence of associated extra-intestinal and intestinal anomalies which make their prognosis worse. [3] Situs inversus, pubic diastasis, bifid clitoris, double vagina, anteriorly placed anus, atrial/ventricular septal defect, patent ductus arteriosus, Choledochal cyst, cerebral arterio-venous malformation, macrocephaly, scoliosis and stenosis of superior vena cava have all been variously reported. [1, 3] Intestinal atresia in gastroschisis is a known association. [3] Hombalker et al. described a case of LSG with caecal agenesis. [4] Our patient had multiple atresias and a perforation in proximal ileum. This worsened sepsis and prognosis in our patient.

**Figure:**
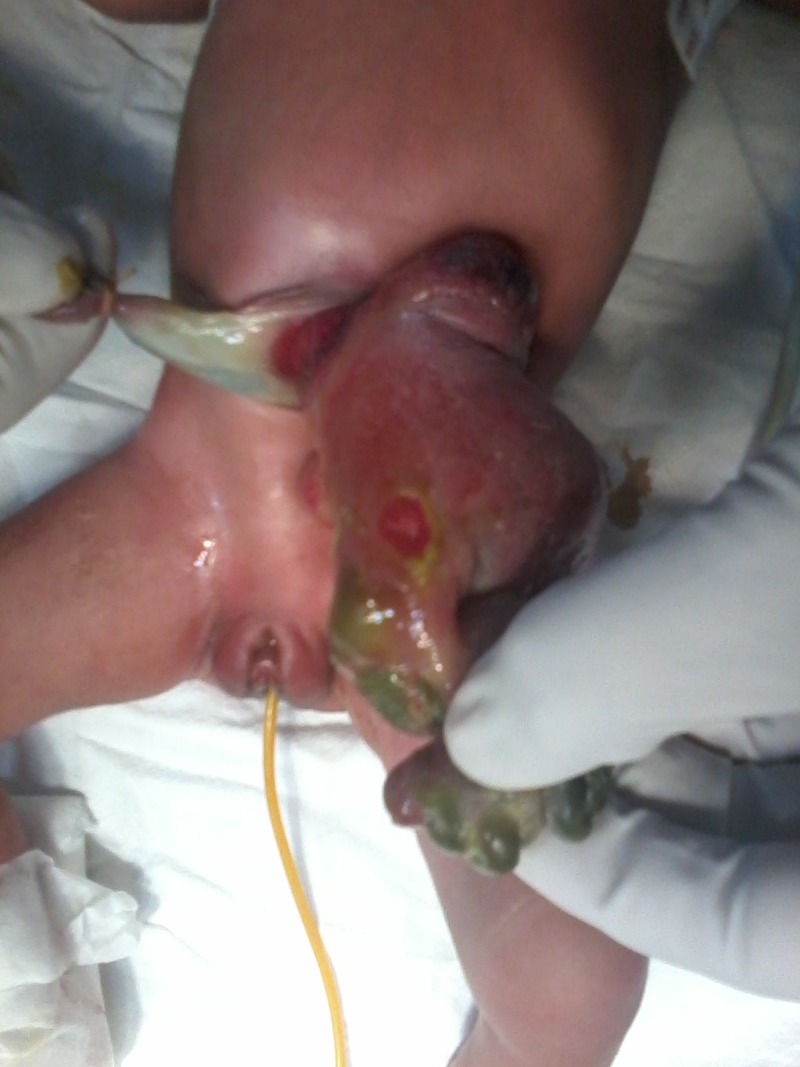
Figure 1: Left sided gastroschisis with intestinal perforation.

## Footnotes

**Source of Support:** Nil

**Conflict of Interest:** None
